# Effectiveness of Split-Thickness Skin Grafting in Nonhealing Ulcers: Impact of Patient Demographics, Comorbidities, and Ulcer Etiology

**DOI:** 10.7759/cureus.95015

**Published:** 2025-10-20

**Authors:** Laxmikantha C, Shilpashree Channasandra Shekar, Madan Haravu Srikantegowda

**Affiliations:** 1 Department of General Surgery, Sri Chamundeshwari Medical College, Hospital and Research Institute, Channapatna, IND

**Keywords:** chronic nonhealing ulcer, full-thickness skin graft, graft integration, skin graft, split-thickness skin graft, wound healing

## Abstract

Objective: The primary aim of the present study was to evaluate the outcomes of split-thickness skin grafting (STSG) in the management of nonhealing ulcers. The secondary aim was to assess the impact of demographic factors, comorbidities, and ulcer etiology on graft take and overall healing outcomes.

Methodology: This longitudinal, cross-sectional study was conducted in the Department of General Surgery, Shivamogga Institute of Medical Sciences, Karnataka, India, between January 2021 and December 2023. Patients aged 20-60 years with nonhealing ulcers of more than three weeks’ duration and without uncontrolled systemic illness were included. The calculated sample size was 80, of whom 52 underwent STSG and were analyzed for graft outcomes. Grafts were harvested from the thigh using standard techniques, and patients were followed for four weeks to assess healing, graft take, and complications. Data were collected using a structured proforma and analyzed with IBM SPSS Statistics for Windows, Version 20 (Released 2011; IBM Corp., Armonk, New York, United States), applying Student’s t-test, one-way analysis of variance (ANOVA), and Chi-square or Fisher’s exact test, with p < 0.05 considered statistically significant.

Results: In the STSG group (N = 52), the most frequent healing durations were five weeks in 23 (28.9%) patients and six weeks in 22 (27.6%) patients, while early healing at three weeks was observed in only two (2.6%) patients. The mean healing times by wound size were 5.2 weeks for ulcers <50 cm², 4.7 weeks for 50-100 cm², and 5.9 weeks for >100 cm², with no statistically significant difference (ANOVA: F = 1.72, p = 0.19). By location, 18 (22.5%) ulcers were on the right foot, 21 (26.3%) on the right leg, 25 (31.3%) on the left foot, and 16 (20.0%) on the left leg, with mean healing times ranging from 4.6 to 5.7 weeks, also without significant difference (ANOVA: F = 1.68, p = 0.18). Age significantly influenced healing, with patients aged <40 years (50, 62.5%) showing a mean healing time of 4.6 weeks compared with 6.1 weeks in those aged >40 years (30, 37.5%) (t = 4.12, p < 0.001). Graft take was <90% in 27 (33.8%) patients, 90-99% in 26 (32.5%), and 100% in 27 (33.8%), with higher graft take associated with significantly shorter healing times (<90% vs. 90-99%: t = 5.08, p < 0.001; 90-99% vs. 100%: t = 2.12, p = 0.039; <90% vs. 100%: t = 3.25, p = 0.002).

Conclusion: Skin grafting provides favorable healing outcomes in chronic nonhealing ulcers, with age, wound size, and graft take percentage significantly influencing healing duration. Optimizing preoperative wound conditions may enhance graft acceptance and reduce healing time.

## Introduction

Skin grafting is a widely used surgical technique for managing chronic ulcers, particularly in the lower extremities, where chronic conditions such as diabetes, venous insufficiency, or trauma often lead to significant tissue loss and impaired healing [1]. Chronic nonhealing ulcers in these regions present a therapeutic challenge due to inadequate vascular supply, mechanical stress, and associated comorbidities, which contribute to prolonged recovery, recurrent infections, and reduced quality of life. The global prevalence of chronic leg ulcers is estimated to range between 1% and 2% in the general population, with higher rates observed among elderly and diabetic individuals, highlighting the substantial burden of this condition [2].

Split-thickness skin grafting (STSG) is generally preferred for wounds with a large surface area because it provides rapid coverage, facilitates re-epithelialization, and demonstrates a relatively high success rate in terms of graft take, wound healing, and reduced recurrence. Reported graft take rates in chronic ulcer management range between 85% and 95% in various studies, reflecting its overall effectiveness [3]. In contrast, full-thickness skin grafts (FTSG) are typically reserved for smaller wounds where superior cosmetic and functional outcomes are desired, though their use is limited by donor site morbidity. The choice between STSG and FTSG depends on ulcer size, depth, location, and the overall health status of patients [3].

For grafting to succeed, the wound bed must be well-prepared, well-vascularized, clean, and infection-free, usually achieved through debridement, negative-pressure wound therapy, or prophylactic antibiotics [4]. Despite these measures, complications such as infection, graft failure, and inadequate vascular integration remain significant concerns, particularly in patients with diabetes, vascular insufficiency, or compromised immunity [4]. Advances in wound care, including bioengineered skin substitutes and growth factor therapies, have shown promise in improving graft outcomes; however, their widespread clinical application remains limited [5].

Although the benefits of STSG are well-established, there is limited evidence evaluating its efficacy across different ulcer etiologies while accounting for patient demographics and comorbidities. This gap restricts optimal patient selection and perioperative management. Therefore, the primary objective of the present study was to evaluate the outcomes of STSG in the management of nonhealing ulcers. The secondary objectives were to assess the influence of demographic factors, comorbidities, and ulcer etiology on graft take and overall healing outcomes.

## Materials and methods

Study design and setting

This prospective observational study was conducted in the Department of General Surgery at Shivamogga Institute of Medical Sciences, a tertiary care center in Karnataka, India, between January 2021 and December 2023. Both outpatient and inpatient cases presenting with nonhealing ulcers were evaluated during this period.

Study population

The study included patients aged 20-60 with nonhealing ulcers on the trunk or limbs who were admitted to the Department of General Surgery and met the eligibility criteria.

Sample size calculation

The minimum required sample size was calculated as 80 using the single-proportion formula:

\[ n = \frac{Z^{2} \times p \times (1 - p)}{d^{2}} \]

where Z = 1.96 (for 95% confidence), p = 0.80 (anticipated success proportion), and d = 0.10 (margin of error). Substituting these values yielded n = 61.4, rounded to 80 to compensate for possible attrition and ensure adequate statistical power. Accordingly, 80 patients were recruited. Based on intraoperative findings and wound bed suitability, 52 patients underwent STSG. They were included in the final analysis of graft outcomes, wound size, ulcer location, and healing time. The remaining patients underwent FTSG, contributing to baseline demographic and complication data. This approach allowed the primary objective of evaluating the effectiveness of STSG to be addressed with methodological transparency.

Eligibility criteria

The inclusion criteria comprised patients aged 20 to 60 with nonhealing ulcers of more than three weeks’ duration, absence of uncontrolled systemic illness, and controlled diabetes when present. The exclusion criteria included patients younger than 20 or older than 60, those with severely infected wounds, suspected malignant ulcers, and individuals requiring reconstructive procedures other than grafting.

Preoperative wound assessment

All patients underwent a standardized preoperative wound evaluation to ensure suitability for grafting. A chronic ulcer was defined as a wound that failed to show significant healing progression after three weeks of appropriate conventional management, including debridement, dressing, and infection control. Each ulcer was assessed for size, depth, location, and the condition of the surrounding skin.

The wound bed was evaluated for granulation tissue, necrosis, and exudate. Clinical signs of infection, such as erythema, purulent discharge, or foul odor, were documented. Where infection was suspected, wound swabs were obtained for culture and sensitivity testing, and targeted antibiotic therapy was provided before grafting. Only wounds demonstrating healthy granulation tissue and absence of active infection were considered ready for graft application.

Ulcer classification and infection assessment

Ulcers were classified according to their predominant underlying etiology, determined through detailed clinical evaluation. Diabetic ulcers were identified in patients with known diabetes and characteristic neuropathic or ischemic changes, even when secondary infection was present. Infective ulcers were lesions occurring in nondiabetic individuals that showed local signs of infection, such as purulent discharge, cellulitis, or abscess formation. Traumatic, burn, and venous ulcers were categorized based on clinical history and examination findings. Ulcers resulting from peripheral arterial disease or critical limb-threatening ischemia were excluded during preoperative assessment, as these were not suitable for grafting due to poor vascularity. Patients with uncontrolled diabetes, defined as fasting blood glucose levels exceeding 200 mg/dL or random blood glucose above 300 mg/dL, were excluded until glycemic control was achieved.

Graft infections were diagnosed clinically based on erythema, warmth, tenderness, purulent exudate, or partial graft detachment. Where indicated, wound swabs were obtained for culture and sensitivity testing to guide antibiotic therapy. 

Surgical procedures

For STSG, grafts were harvested from the thigh using a Humby’s knife under aseptic precautions and general or regional anesthesia. Grafts were soaked in saline, meshed to increase surface area and drainage, and applied to the prepared wound bed. The grafted site was dressed with sterile Vaseline gauze, and the donor site with epinephrine-soaked gauze. Dressings were removed on the fifth postoperative day, and patients were followed for four weeks.

For FTSG, full-thickness grafts were harvested from donor sites such as the groin or supraclavicular region. Donor sites were primarily closed, while grafts were sutured to the recipient site under tension-free approximation and secured with bolster dressings.

Data collection

Ethical approval was obtained from the Institutional Ethics Committee (SIMS/IEC/537/2020-21), and written informed consent was taken from all participants in English or Kannada. Data were collected using a structured proforma that included demographic details, comorbidities, ulcer etiology, clinical examination, wound characteristics, and laboratory investigations (complete blood count, random blood sugar, renal and liver function tests, and wound culture). Follow-up was conducted weekly for four weeks to monitor healing, graft take, and complications.

Statistical analysis

Descriptive statistics were used to summarize demographic and clinical characteristics of the whole cohort of 80 patients. Analyses of graft outcomes, including healing time, wound size, ulcer location, graft take, and complications, were restricted to the STSG subgroup of 52 patients in line with the study objective. Quantitative variables were expressed as mean ± standard deviation, and qualitative variables as frequency and percentage. Comparisons across groups were made using Student’s t-test or one-way ANOVA for continuous variables, and Chi-square or Fisher’s exact test for categorical variables. A p-value <0.05 was considered statistically significant. Analyses were performed using IBM SPSS Statistics for Windows, Version 20 (Released 2011; IBM Corp., Armonk, New York, United States), and graphical outputs were generated in MS Excel (Microsoft Corporation, Redmond, Washington, United States).

## Results

Table 1 shows the distribution of patients (N = 80) according to age group, gender, BMI, and ulcer etiology. The age distribution was as follows: 20-30 years, 26 (32.5%); 31-40 years, 24 (30%); 41-50 years, 15 (18.75%); and 51-60 years, 15 (18.75%). The cohort comprised 42 (52.5%) males and 38 (47.5%) females. For BMI, 32 (40%) patients were of normal weight, while 48 (60%) were overweight or obese. With respect to etiology, 32 (40%) cases were infective, 27 (33.75%) diabetic, 11 (13.75%) trauma-related, eight (10%) burn-related, and two (2.5%) venous in origin. Significant differences were observed for age group (p = 0.0155) and etiology (p < 0.0001), whereas gender (p = 0.65) and BMI (p = 0.07) were not statistically significant.

**Table 1:** Distribution of age group, gender, BMI, and etiology of patients BMI: body mass index; * shows significant p-value; (a) mean BMI (normal group): 23.2 kg/m² (range: 18.5-24.9); (b) mean BMI (overweight/obese group): 31.2 kg/m² (range: 25.0-38.7) p-value < 0.05 was considered significant

Variables	Total number (N = 80) N (%)	Chi-square	p-value
Age group		10.4	0.0155*
20 to 30 years	26 (32.5%)		
31 to 40 years	24 (30%)		
41 to 50 years	15 (18.75%)		
51 to 60 years	15 (18.75%)		
Gender		0.2	0.65
Male	42 (52.50%)		
Female	38 (47.5%)		
BMI		3.2	0.07
Normal^a^	32 (40%)		
Overweight/obese^b^	48 (60%)		
Ulcer etiology		4.71	<0.0001*
Trauma	11 (13.75%)		
Burn	8 (10%)		
Infective	32 (40%)		
Diabetic	27 (33.75%)		
Venous	2 (2.50%)		

STSG was performed in 52 (65%) patients, while 28 (35%) received FTSG. Graft outcomes were almost equally distributed, with 27 (33.75%) patients showing <90% uptake, 26 (32.5%) patients achieving 90-99% uptake, and 27 (33.75%) patients achieving complete uptake. Postgraft complications were infrequent, occurring as infection in five (6.25%) patients and total graft failure (no uptake) in four (5%) patients, while 71 (88.75%) patients had no complications. Statistically significant associations were observed for graft type (p = 0.0073) and postoperative complications (p < 0.0001), whereas graft uptake outcomes did not differ significantly (p = 0.98) (Table 2).

**Table 2:** Distribution according to type of graft, outcomes, and postgraft complication (N = 80) STSG: split-thickness skin grafting; FTSG: full-thickness skin grafts; * shows a significant p-value Outcomes represent the percentage of graft take after the procedure, where <90% indicates less than 90% graft take, 90-99% indicates 90% to 99% graft take, and 100% indicates complete graft take

Parameters	Total number N (%)	Chi-square	p-value
Type of graft		7.2	0.0073*
STSG	52 (65%)		
FTSG	28 (35%)		
Outcomes		0.025	0.98
<90%	27 (33.75%)		
90-99%	26 (32.5%)		
100%	27 (33.75%)		
Complications (postgraft)		110.4	<0.0001*
Infections	5 (6.25%)		
No graft uptake	4 (5%)		
None	71 (88.75%)		

**Figure 1:**
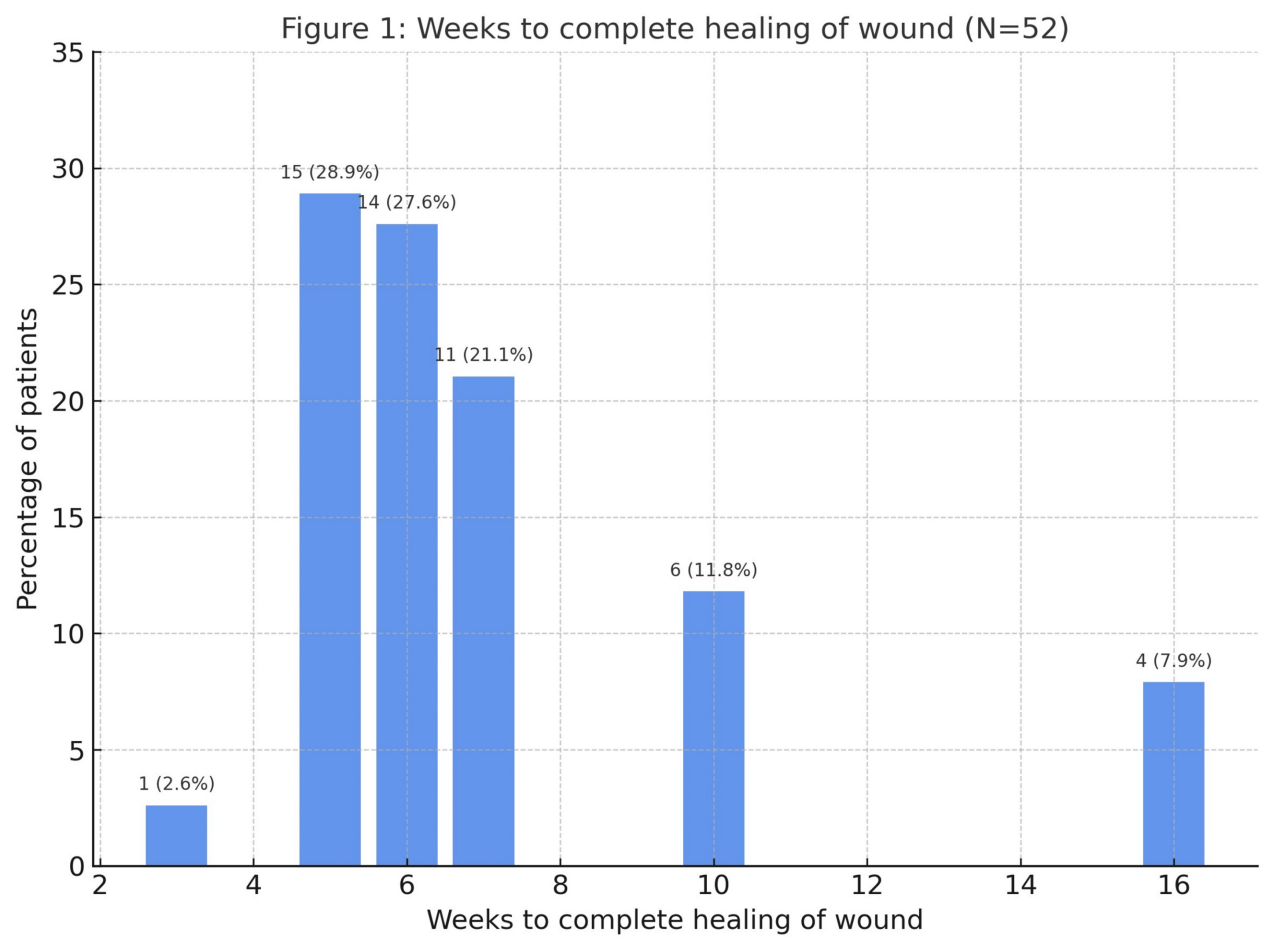
Time duration (weeks) to complete healing of wound in split-thickness skin grafting (N = 52)

The most common healing times were five weeks in 23 (28.9%) patients and six weeks in 22 (27.6%). Healing at seven weeks occurred in 17 (21.1%) patients, while 10 (11.8%) healed by 10 weeks and six (7.9%) by 16 weeks. Early healing at three weeks was least frequent, observed in two (2.6%) patients (Figure 1).

Wound sizes were <50 cm² in 23 (28.8%) patients, 50-100 cm² in 35 (43.8%), and >100 cm² in 22 (27.5%), with corresponding mean healing times of 5.2, 4.7, and 5.9 weeks, respectively. The difference in healing times across wound size categories was insignificant (ANOVA F = 1.72, p = 0.19). With respect to wound location, 18 (22.5%) ulcers were on the right foot, 21 (26.3%) on the right leg, 25 (31.3%) on the left foot, and 16 (20.0%) on the left leg, with mean healing times ranging from 4.6 to 5.7 weeks. No statistically significant difference was observed among locations (ANOVA F = 1.68, p = 0.18) (Table 3).

**Table 3:** Wound size and location with respect to mean weeks to healing in split-thickness skin grafting group (N = 52) ANOVA: analysis of variance

Parameters	Number N (%)	Mean weeks to healing	ANOVA value (F)	p-value
Wound size			1.72	0.19
Wound size <50 sq cm	23 (28.75%)	5.2		
Wound size 50-100 sq cm	35 (43.75%)	4.7		
Wound size >100 sq cm	22 (27.50%)	5.9		
Wound location			1.68	0.18
Right foot	18 (22.5%)	5.1		
Right leg	21 (26.25%)	4.6		
Left foot	25 (31.25%	5.5		
Left leg	16 (20%)	5.7		

Patients aged <40 years comprised 50 (62.5%) with a mean healing time of 4.6 weeks, while those >40 years accounted for 30 (37.5%) with a mean healing time of 6.1 weeks. The difference in healing duration between the two age groups was statistically significant (unpaired t = 4.12, p < 0.001). With respect to graft take, 27 (33.8%) patients had <90% take with a mean healing time of 7.9 weeks, 26 (32.5%) had 90-99% take with a mean healing time of 5.1 weeks, and 27 (33.8%) achieved 100% graft take with a mean healing time of 4.7 weeks. Age groups (<40 vs ≥40 years) were compared using an unpaired t-test, which showed significantly longer healing time in patients ≥40 years (t = 4.12, p < 0.001). Percent graft take was analyzed using one-way ANOVA, which revealed a significant difference in healing times among the three groups (F = 10.42, p < 0.001). Post hoc Tukey’s HSD analysis demonstrated that patients with <90% graft take had significantly longer healing times than 90-99% and 100% graft take groups (p < 0.001 and p = 0.002, respectively). Additionally, healing time differed significantly between the 90-99% and 100% graft take groups (p = 0.039) (Table 4).

**Table 4:** Distribution of patients and mean healing time by age and graft take percentage in split-thickness skin grafting group (N = 52) t: unpaired t-test value; A: ANOVA value; ANOVA: analysis of variance; * shows a significant p-value A p-value of <0.05 was considered significant. Outcomes represent the percentage of graft take after the procedure, where <90% indicates less than 90% graft take, 90-99% indicates 90% to 99% graft take, and 100% indicates complete graft take

Parameters	Total number N (%)	Mean weeks to healing	Test values	p-value
Age			4.12^t^	<0.001*
20 to 40 years	50 (62.5%)	4.6		
40 to 60 years	30 (37.5%)	6.1		
Outcomes			10.42^A^	<0.001*
<90%	27 (33.75%)	7.9		
90-100%	26 (32.5%)	5.1		
100%	27 (33.75%	4.7		

## Discussion

This study evaluated the demographic characteristics, etiological factors, surgical interventions, and postoperative outcomes of patients undergoing STSG or FTSG for chronic nonhealing ulcers. Most patients were aged 20-40 years, which aligns with similar findings reported by Mohan et al. [6], who noted that chronic ulcers significantly affect younger, economically active populations, not only the elderly. The male predominance in our study is also consistent with findings reported by Mohan et al. [6], who identified increased trauma and burn exposure among males as significant contributors.

Overweight or obesity was present in 60% of patients, reflecting growing evidence that obesity impairs wound healing through microcirculatory dysfunction and chronic inflammation. Similar findings were reported by Donegan et al. [7], where diabetic patients, often overlapping with obese populations, had delayed healing by approximately two weeks compared with nondiabetics.

The prevalence of infective (40%) and diabetic (33.75%) etiologies was dominant in our cohort, consistent with findings reported by Mohan et al. [6], where diabetic foot ulcers and infections were the leading causes of chronic wounds. STSG was the preferred graft type in 65% of cases, reflecting its adaptability and favorable graft take. Similar findings were reported by Serra et al. [8], who considered autologous STSG the gold standard for chronic leg ulcers.

Improved graft uptake in our study correlated with faster healing, consistent with findings reported by Rajavelu et al. [9], who demonstrated an inverse relationship between graft uptake and healing duration. Postoperative complications were minimal, with infection in 6.25% and graft failure in 5%, while 88.75% experienced uneventful recovery. Similar findings were reported by Anderson et al. [10], who documented low complication rates, including a 2.8% infection rate with STSG and an average healing time of five weeks.

Healing in our study generally occurred within 5-6 weeks, although some patients required up to 16 weeks. Similar findings were reported by Rose et al. [11], where STSG in high-risk diabetic foot patients achieved complete healing in 69% of cases. Healing duration was influenced by wound size, patient age, location, and graft uptake. Larger wounds (>100 cm²) and age >40 years were associated with delayed healing, consistent with biological principles of reduced regenerative capacity in older patients [6].

Our results also emphasize that optimal graft absorption strongly predicts faster healing. Turissini et al. [12] reported similar findings, where bacterial colonization, particularly with *Pseudomonas aeruginosa*, significantly reduced graft success. A Danish study by Høgsberg et al. [13] reported that only 33% of *Pseudomonas*-positive ulcers healed by week 12, compared with 73% of *Pseudomonas*-negative ulcers.

Previous studies have explored adjunctive therapies such as dermal regeneration templates (DRT) and negative pressure wound therapy (NPWT), which have demonstrated beneficial effects in improving graft outcomes. For instance, Rohrich et al. [14] reported that DRT use before STSG reduced graft failure (5.2% vs. 19.2%) and improved ambulation, while Turissini et al. [12] found that NPWT reduced STSG failure by nearly 80% compared with conventional dressings. However, these findings are derived from prior literature; in the present study, only STSG was evaluated without using adjunctive therapies.

The demographic findings of our study mirror those reported by Haris et al. [15], where most patients were 20-40 years old and engaged in high-risk occupations. Male predominance (52.5%) was consistent with findings reported by Rajavelu et al. [9], who observed 65.3% male cases in their prospective study. The high prevalence of obesity (60%) is also consistent with findings reported by Pierpont et al. [16] and Cotterell et al. [17], who highlighted impaired angiogenesis, poor perfusion, and chronic inflammation as mechanisms of delayed healing. Similar findings were reported by Mizuo et al. [18], who emphasized that reduced capillary density and fibrotic adipose tissue hinder revascularization, thereby increasing graft failure risk.

Infective (40%) and diabetic (33.75%) etiologies were also consistent with findings reported by Rajavelu et al. [9], who identified diabetic and venous origins as common causes. The preference for STSG in 65% of cases reflects its recognized role as a practical option for chronic leg ulcers, with similar findings reported by Braza et al. [19]. Faster healing with improved graft uptake in our patients was also consistent with findings reported by Braza et al. [19], who demonstrated an inverse relationship between graft take and healing time.

In our study, complications were limited, with infections in 6.25% and graft failure in 5%, consistent with findings reported by Serra et al. [8], who emphasized the importance of proper wound bed preparation and perioperative care. Healing times of 5-6 weeks, extending up to 16 weeks, are also consistent with findings reported by Rose et al. [11] in chronic ulcer populations. Advanced age, larger wounds, and reduced graft uptake were associated with prolonged healing, consistent with the findings of Haris et al. [15].

A recent prospective cohort study in Yemen confirmed that postoperative infection, particularly with gram-negative bacteria, was the strongest independent predictor of STSG failure (odds ratio = 48) [20]. Turissini et al. [12] reported similar findings, where NPWT significantly reduced failure compared with bolster dressings, while bacterial contamination and comorbidities such as congestive heart failure increased failure risk.

Our findings support the effectiveness of STSG for chronic nonhealing ulcers, provided that patient comorbidities (diabetes, obesity, infection) and wound-related factors (size, location, microbial colonization) are optimized. Adjunctive techniques such as DRT and NPWT further enhance outcomes, while *Pseudomonas* colonization and systemic illness, such as heart failure, compromise success. These results underscore the importance of thorough preoperative evaluation, strict infection control, metabolic stabilization, and using graft-supportive adjuncts to maximize success.

Limitations 

This study has several limitations that should be considered when interpreting the findings. First, it was a single-center observational study conducted in a tertiary care setting, which may limit the generalizability of the results to other populations and healthcare environments. Second, the sample size for STSG analysis was relatively small (52 patients), and the sample size calculation had minor inconsistencies, both of which may have reduced the statistical power and precision of specific estimates. Third, the follow-up period was limited to four weeks, restricting the evaluation of long-term outcomes such as graft durability, ulcer recurrence, and functional recovery. Fourth, the nonrandomized design may have introduced selection bias and limited the ability to infer causality. Finally, microbiological profiling was limited, and antibiotic resistance patterns, which could influence graft success, were not comprehensively studied.

Despite these limitations, the study provides valuable insights into the clinical effectiveness of STSG in nonhealing ulcers and highlights the demographic and wound-related factors that influence healing outcomes.

## Conclusions

This study demonstrates that STSG is an effective and reliable technique for managing nonhealing foot and leg ulcers, particularly those associated with diabetes. STSG provides adequate wound coverage, facilitates healing, and contributes to improved functional outcomes. Our findings are consistent with similar findings reported in the literature, which support STSG as a preferred option due to its simplicity, cost-effectiveness, and high graft take rates when patient-specific risk factors, including infection control, vascular status, nutritional status, and comorbidities such as diabetes and obesity, are appropriately managed. Careful preoperative optimization and structured postoperative care can enhance graft uptake, improve durability, reduce recurrence, and improve patient quality of life.

## References

[1] Ghaly P, Iliopoulos J, Ahmad M (2021). The role of nutrition in wound healing: an overview. Br J Nurs.

[2] Deng X, Gould M, Ali MA (2022). A review of current advancements for wound healing: biomaterial applications and medical devices. J Biomed Mater Res B Appl Biomater.

[3] Singh H, Sharma A (2025). Evaluating the efficacy of skin grafting in accelerating healing of lower extremity ulcers: efficacy of skin grafting in lower extremity ulcers. ACMR.

[4] Kim PJ, Evans KK, Steinberg JS, Pollard ME, Attinger CE (2013). Critical elements to building an effective wound care center. J Vasc Surg.

[5] Lane KL, Abusamaan MS, Voss BF (2020). Glycemic control and diabetic foot ulcer outcomes: a systematic review and meta-analysis of observational studies. J Diabetes Complications.

[6] P AA, Mohan A, S KB (2022). Study of efficacy of skin grafting in healing lower extremity ulcers in a rural tertiary care hospital. Int Surg J.

[7] Donegan RJ, Schmidt BM, Blume PA (2014). An overview of factors maximizing successful split-thickness skin grafting in diabetic wounds. Diabet Foot Ankle.

[8] Serra R, Rizzuto A, Rossi A (2017). Skin grafting for the treatment of chronic leg ulcers-a systematic review in evidence-based medicine. Int Wound J.

[9] Rajavelu N, Devi K, Rajam V (2024). Outcome of split thickness skin graft for the treatment of non-healing foot and leg ulcers: a prospective study. Int J Pharm Clin Res.

[10] Anderson JJ, Wallin KJ, Spencer L (2012). Split thickness skin grafts for the treatment of non-healing foot and leg ulcers in patients with diabetes: a retrospective review. Diabet Foot Ankle.

[11] Rose JF, Giovinco N, Mills JL, Najafi B, Pappalardo J, Armstrong DG (2014). Split-thickness skin grafting the high-risk diabetic foot. J Vasc Surg.

[12] Turissini JD, Elmarsafi T, Evans KK, Kim PJ (2019). Major risk factors contributing to split thickness skin graft failure. Georgetown Med Rev.

[13] Høgsberg T, Bjarnsholt T, Thomsen JS, Kirketerp-Møller K (2011). Success rate of split-thickness skin grafting of chronic venous leg ulcers depends on the presence of Pseudomonas aeruginosa: a retrospective study. PLoS One.

[14] Rohrich RN, Li KR, Lava CX (2025). Outcomes of dermal regeneration template and split-thickness skin grafts in lower extremity wound closure: an 8-year retrospective analysis. Wounds.

[15] Haris S, Nema PK, Charokar K, Gupta N (2025). A clinical study of the role of split-thickness skin autograft in management of wounds and identification of factors influencing the graft uptake. Int Surg J.

[16] Pierpont YN, Dinh TP, Salas RE, Johnson EL, Wright TG, Robson MC, Payne WG (2014). Obesity and surgical wound healing: a current review. ISRN Obes.

[17] Cotterell A, Griffin M, Downer MA, Parker JB, Wan D, Longaker MT (2024). Understanding wound healing in obesity. World J Exp Med.

[18] Mizuo C, Garwood CS (2017). Managing lower extremity wounds in obese patients. Podiatr Today.

[19] Braza ME, Marietta M, Fahrenkopf MP (2025). Split-Thickness Skin Grafts. https://www.ncbi.nlm.nih.gov/books/NBK551561/.

[20] Abdulmughni LA, Al-Sanabani JA, Gilan WM, Issa MA, Jowah HM (2025). Split-thickness skin graft outcomes and associated risk factors in patients with skin defects at Al-Gumhouri hospital, Sana'a, Yemen: a prospective observational study. BMC Surg.

